# MRAP2 Interaction with Melanocortin-4 Receptor in SnakeHead (*Channa argus*)

**DOI:** 10.3390/biom11030481

**Published:** 2021-03-23

**Authors:** Zheng-Yong Wen, Ting Liu, Chuan-Jie Qin, Yuan-Chao Zou, Jun Wang, Rui Li, Ya-Xiong Tao

**Affiliations:** 1 Key Laboratory of Sichuan Province for Fish Conservation and Utilization in the Upper Reaches of the Yangtze River, Neijiang Normal University, Neijiang 641100, China; qinchuanjie@126.com (C.-J.Q.); zou3891@163.com (Y.-C.Z.); wangjunzl@126.com (J.W.); liruitiandi@sina.com (R.L.); 2 College of Life Science, Neijiang Normal University, Neijiang 641100, China; 3 BGI Education Center, University of Chinese Academy of Sciences, Shenzhen 518083, China; 4 Department of Anatomy, Physiology and Pharmacology, College of Veterinary Medicine, Auburn University, Auburn, AL 36849, USA; tzl0057@auburn.edu

**Keywords:** *Channa argus*, MC4R, MRAP2, distribution, pharmacology

## Abstract

The melanocortin-4 receptor (MC4R) plays an important role in the regulation of food intake and energy expenditure. Melanocortin-2 receptor accessory protein 2 (MRAP2) modulates trafficking, ligand binding, and signaling of MC4R. The Northern snakehead (*Channa argus*) is an economically important freshwater fish native to East Asia. To explore potential interaction between snakehead MC4R and MRAP2, herein we cloned snakehead *mc4r* and *mrap2*. The snakehead *mc4r* consisted of a 984 bp open reading frame encoding a protein of 327 amino acids, while snakehead *mrap2* contained a 693 bp open reading frame encoding a protein of 230 amino acids. Synteny analysis indicated that *mc4r* was highly conserved with similar gene arrangement, while *mrap2* contained two isoforms in teleost with different gene orders. Snakehead *mc4r* was primarily expressed in the brain, whereas *mrap2* was expressed in the brain and intestine. Snakehead *mc4r* and *mrap2* expression was modulated by fasting and refeeding. Further pharmacological experiments showed that the cloned snakehead MC4R was functional, capable of binding to peptide agonists and increasing intracellular cAMP production in a dose-dependent manner. Snakehead MC4R exhibited high constitutive activity. MRAP2 significantly decreased basal and agonist-stimulated cAMP signaling. These findings suggest that snakehead MC4R might be involved in energy balance regulation by interacting with MRAP2. Further studies are needed to elucidate MC4R in regulating diverse physiological processes in snakehead.

## 1. Introduction

The melanocortin-4 receptor (MC4R), a Family A G protein-coupled receptor (GPCR), plays a central role in energy homeostasis regulation in mammals [1,2]. It consists of the hallmark seven transmembrane domains (TMDs) connected by alternating extracellular and intracellular loops, with an extracellular N-terminus and intracellular C-terminus [3]. Since the human MC4R (hMC4R) was first cloned in 1993 by degenerate PCR [3], extensive studies have been done to elucidate the potential physiological and pathophysiological relevance of this receptor. Cone and colleagues showed that MC4R is highly expressed in neuroendocrine and autonomic control circuits in the brain [4]. In 1997, it was discovered that modulation of MC4R activation or genetic inactivation exerts a profound influence on food intake and energy expenditure [5,6,7]. Subsequent studies found that mutations in *MC4R* and common genetic variation near *MC4R* lead to a dominant form of obesity and insulin resistance [8,9,10,11]. Moreover, several studies suggested that MC4R is also involved in modulating the reproductive function of animals [12,13,14].

Activation of neurons expressing pro-opiomelanocortin (POMC) decreases food intake, whereas activation of neurons expressing neuropeptide-Y (NPY) and agouti-related peptide (AgRP) increases food intake [2]. MC4R mediates the agonist signal provided by the POMC-derived peptide α-melanocyte stimulating hormone (α-MSH), and the antagonist signal provided by AgRP [1,15]. After ligand binding, MC4R is primarily coupled to G_s_, which stimulates adenylyl cyclase activity and increases intracellular cAMP level. This classical G_s_-cAMP signaling pathway is required for regulation of energy balance, thermogenesis, and glucose metabolism [16]. Moreover, the pharmacological properties of MC4R are modulated by melanocortin-2 receptor accessory proteins (MRAPs) [17,18].

MRAP family consists of two members, MRAP1 and MRAP2. These two small single transmembrane proteins have been identified to be involved in the regulation of MCR functions as MRAP1 is responsible for the formation of a functional MC2R, and MRAP2 is relevant for the regulation of energy balance [19]. Nonsynonymous *MRAP2* variants have been detected in obese patients, but not in normal weight controls in a meta-analysis [20], and *Mrap2*-deletion leads to obesity in mice [21]. One important mechanism is that MRAP2 exerts its effects on energy homeostasis through action on the MC4R. For classical G_s_-cAMP signaling pathway, MRAP2 does not affect basal activity, but decreases agonist-stimulated cAMP production in human (h) MC4R [22], while MRAP2 suppresses basal signaling, but increases agonist-stimulated maximal response in mouse MC4R [21]. MRAP2 also enhances MC4R-mediated cAMP production in feline [23]. An in vivo experiment showed that MRAP2 potentiates mouse MC4R activation in regulating energy homeostasis at baseline conditions in paraventricular nucleus (PVN) MC4R-expressing neurons [24].

MC4R has been investigated in several non-mammalian vertebrates including ectotherm fish, such as goldfish [25], rainbow trout [26], spotted scat [27], grass carp [28], ricefield eel [29], orange-spotted grouper [30], and topmouth culter [31]. Similar to mammalian MC4Rs, teleost MC4R is also involved in regulating food intake and maintaining energy balance. In barfin flounder, food deprivation induces significantly higher *mc4r* expression in the liver [32], and the same phenomenon was observed in the brain of Ya-fish [33]. Meanwhile, intracerebroventricular administration of MC4R agonist melanotan II inhibits food intake, whereas intracerebroventricular injection of selective MC4R antagonist HS024 stimulates food intake in goldfish [25]. In addition, nonsynonymous mutations in cavefish *mc4r* were found to play an important role in adapting to nutrient-poor condition [34].

MRAP2 and its modulatory role on MC4R have also been studied in several fishes. Different from mammals, some teleosts, such as zebrafish and topmouth culter, possess two isoforms of MRAP2 [35,36,37], while other lower vertebrates including tilapia, sea lamprey, and grouper, have only one [30,38,39], suggesting that the phylogenetic process in fish may be more complex than in mammals. In addition, MRAP2 has different effects on MC4R expression, ligand binding, and signaling in different fishes. In sea lamprey, MRAP2 interacts with MCRa and MCRb, reducing their expressions on the cell surface, but increases α-MSH-induced signaling of receptors [38]. In tilapia, MRAP2 decreases both cell surface expression and ligand-stimulated signaling at the G_s_-cAMP pathway [39]. In grouper, both basal and agonist-stimulated G_s_-cAMP signaling of MC4R is decreased by MRAP2 [30]. In zebrafish, MRAP2a decreases α-MSH binding affinity, whereas MRAP2b enhances ligand sensitivity of MC4R [35]. In topmouth culter, MRAP2a increases the maximal binding (B_max_) and decreases agonist-induced cAMP, but MRAP2b does not affect B_max_ and agonist-stimulated cAMP [31]. Therefore, more studies about interactions between MRAP2 and MC4R are worth pursuing.

The northern snakehead (*Channa argus*) belongs to the family Channidae and the order Perciformes, and is an economically important freshwater fish native to East Asia [40]. Although the annual production has reached 510,000 tons per year in China, the genetic degradation caused by inbreeding of *C. argus* cultivation has led to higher susceptibility to diseases in recent years [41]. As MC4R is functionally related to growth and reproduction in fish, studies on MC4R will undoubtedly be helpful for understanding the growth traits of *C. argus* and might also be of benefit for future breeding projects. We herein investigated the molecular cloning, tissue distribution, pharmacological characteristics of caMC4R and its regulation by caMRAP2, and a potential role in food intake regulation.

## 2. Materials and Methods

### 2.1. Fish Sampling

The northern snakehead, weight (71.3 ± 5.6 g) used in this study, were obtained from Neijiang Fish Farm (Neijiang, China) and transported to the experimental aquarium at the College of Life Sciences of Neijiang Normal University. The fish were kept in 100 L tanks under natural light-dark conditions (12 L/12 D) with a constant flow of filtered water and water temperature maintained at 18–20 °C. The fish were fed with fish meat (5–8% of body weight) at 19:00 every day. Fish were acclimated for 2 weeks before the experiment, showing a normal feeding pattern during this acclimation period.

Following acclimation, six fish were randomly selected for cloning and tissue distribution studies. The fish were anesthetized on ice and sacrificed by decapitation. Tissue samples including adipose, brain, eye, gill, gonad (ovary), heart, intestine, kidney, liver, muscle, and spleen, were collected and frozen in liquid nitrogen immediately. For a short-term food deprivation experiment, fish were sampled at 19:00 (after feeding, 0 h), 22:00 (3 h), 01:00 (6h), 07:00 (12 h), and 19:00 (24 h). Six fish were selected from each group, and their brains were sampled and stored. For a long-term food deprivation experiment, fish were assigned to 4 experimental tanks (with 20 fish per tank, 2 tanks of fish were fed, and the other 2 tanks were unfed) for two weeks. Five fish were chosen, and their brains were sampled from each group. Subsequently, the fasted group was refed and sampled. All samples were kept at −80 °C until further analysis.

All animal experiments were performed with the approval of the Neijiang Normal University Animal Care and Use Committee and in full compliance with its ethics guidelines.

### 2.2. Ligands and Plasmids

NDP-MSH was purchased from Peptides International (Louisville, KY, USA), α- and β-MSHs were purchased from Pi Proteomics (Huntsville, AL, USA), and ACTH (1–24) was purchased from Phoenix Pharmaceuticals (Burlingame, CA, USA). Both [^125^I]-NDP-MSH and [^125^I]-cAMP were iodinated in our lab using chloramine T method [42,43]. The N-terminal c-myc-tagged hMC4R subcloned into pcDNA3.1 vector was prepared as described in our previous study [44]. The coding sequences of snakehead MC4R and MRAP2 were commercially synthesized and subcloned into pcDNA3.1 vector by GenScript (Piscataway, NJ, USA) to generate the plasmids used for transfection.

### 2.3. Molecular Cloning of Snakehead mc4r and mrap2 Genes

Protein similarity-based blast was performed to search the target genes in the snakehead genome and transcriptome databases [41], using zebrafish *mc4r*, *mrap2a*, and *mrap2b* as references. After searching, we obtained the genomic and transcriptomic sequences of the snakehead *mc4r* and *mrap2* (single copy, showing close relationship with zebrafish *mrap2b*) genes. Two pairs of primers were designed to validate the two sequences, respectively. Total RNA was isolated from the whole brain with the Trizol reagent (Invitrogen, Carlsbad, CA, USA) according to manufacturer’s protocol, and 1 μg of the RNA was reverse transcribed to cDNA using Super ScriptTM II RT reverse transcriptase (Takara, Dalian, China). The basic cycling conditions of the PCR were set as follows: A denaturing stage at 94 °C for 30 s, gene-specific annealing temperature for 45 s and an elongation stage at 72 °C for 60 s, a total of 34 cycles. The PCR products were purified from agarose gel using the Universal DNA Purification Kit (TIANGEN, Beijing, China), and then cloned into the pMD-19T vector (TaKaRa). The inserts were sequenced at BGI (Beijing, China), and the primers used to validate *mc4r* and *mrap2* genes were listed in Table S1.

### 2.4. Sequence Analysis and Data Processing

The coding sequences (CDS) were determined using online software ORF finder (https://www.ncbi.nlm.nih.gov/gorf/gorf.html), and then the putative protein sequences were translated with Primer Premier 5.0 software. Moreover, the Bioedit software was used to determine the isoelectric point (PI), the online tool TMHMM Server v. 2.0 (http://www.cbs.dtu.dk/services/TMHMM-2.0/) was used to identify the transmembrane domain, and the Expasy (http://www.expasy.org/tools/) was used to predict glycosylation and phosphorylation sites. Furthermore, multiple sequence alignments were done as described in our previous work [45], and the predicted secondary structure of snakehead MC4R was drawn according to two related studies [2,34]. Additionally, three dimensional structures of several vertebrate MC4Rs were predicted with an online tool SWISS-MODEL (https://www.swissmodel.expasy.org/) using human MC4R as reference [46]. Finally, a comparative genomic survey was performed to investigate the gene synteny of *MC4R* and *MRAP2* in vertebrates as described previously [47,48], based on a series of representative animal genomes.

### 2.5. Phylogenetic Analysis

The predicted snakehead MC4R and MRAP2 protein sequences together with those of other vertebrates were aligned by CLUSTAL X2.1, respectively. The aligned protein datasets were used to construct the phylogenetic tree with Neighbor-Joining approach using Mega 6.0 software [49]. The best-fitting model was calculated by MrmodelTest 2.0 [50] and ProtTest 2.4 [51], and the JTT + G model was selected as the best model after evaluation. The phylogenetic trees were redrawn using Figtree software. The robustness of the tree topology was assessed by nonparametric bootstrap analysis with 1000 resampling replicates. IDs of the protein sequences used in present study are shown in Tables S2 and S3.

### 2.6. Quantitative Real-Time PCR

Extraction of total RNA from fish tissues and first strand cDNA synthesis were performed as described above. Then, real-time PCR was used to detect the mRNA expressions of *mc4r* and *mrap2* with LightCycler Real-Time system. Reverse transcription product was used for real-time PCR in a final volume of 10 μL. The end products of PCR were verified with the melting curves that showing a single peak specific for the target gene. The relative expression levels were calculated according to the method described previously [52], and *tubα1* was used as reference gene. Primers used to amplify *mc4r*, *mrap2*, and *tubα1* are shown in Table S1. Values are expressed as means ± SEM (*n* = 6).

### 2.7. Cell Culture and Transfection

Human Embryonic Kidney (HEK) 293T cells, purchased from American Type Culture Collection (Manassas, VA, USA), were cultured in Dulbecco’s Modified Eagle’s medium (DMEM) (Invitrogen) containing 10% newborn calf serum (PAA Laboratories, Etobicoke, ON, Canada), 10 mM HEPES, 0.25 μg/mL of amphotericin B, 50 μg/mL of gentamicin, 100 IU/mL of penicillin, and 100 μg/mL of streptomycin at 37 °C in a 5% CO_2_-humidified atmosphere. The cells were plated into 6-well or 24-well plates (Corning, NY, USA) pre-coated with 0.1% gelatin and transfected or co-transfected when cells reached at ~70% confluence using calcium phosphate precipitation method [53].

### 2.8. Flow Cytometry Assay

To investigate the effect of snakehead MRAP2 on the cell surface and total expression levels of MC4R, cells were plated into 6-well plates and transfected with snakehead MC4R and MRAP2 plasmids in four ratios (1:0, 1:1, 1:3, and 1:5). Then, cells were incubated with mouse anti-myc 9E10 monoclonal antibody (Developmental Studies Hybridoma Bank, The University of Iowa, Iowa City, IA, USA) diluted 1:40 and Alexa Fluor 488-labeled goat anti-mouse antibody (Invitrogen) diluted 1:2000 for 1 h, respectively. Flow cytometry was carried out, and C6 Accuri Cytometer (Accuri Cytometers, Ann Arbor, MI, USA) was applied to collect fluorescence signals. The expression level of caMC4R (caMC4R fluorescence − pcDNA3.1 fluorescence) from 1:0 (caMC4R/caMRAP2) group was set as 100%, and the expression levels of other groups were calculated as a percentage of 1:0 group [54].

### 2.9. Ligand Binding Assays

To assess the ligand binding properties of the caMC4R, four ligands (NDP-MSH, ACTH (1–24), α-MSH, β-MSH) were used. Forty-eight hours after transfection, HEK293T cells were washed twice with warm DMEM containing 1 mg/mL bovine serum albumin (BSA, EMD Millipore Corporation, Billerica, MA, USA) (DMEM/BSA). To study the effects of caMRAP2 on the binding property of caMC4R, two ligands (α-MSH and ACTH (1–24)) were used. Forty-eight hours after transfection, HEK293T cells co-transfected with caMC4R and caMRAP2 at four ratios (1:0, 1:1, 1:3, and 1:5) were washed twice with warm DMEM/BSA. After 1-h incubation with ligands, the competitive binding reaction was terminated by washing cells twice with cold Hank’s balanced salt solution containing 1 mg/mL BSA on ice. The cells were lysed with 0.5 M NaOH, collected with cotton swabs, and radioactivity detected with gamma counter (Cobra II Auto-Gamma, Packard Bioscience, Frankfurt, Germany).

### 2.10. cAMP Assays

Forty-eight hours after transfection, HEK293T cells were washed twice with warm DMEM/BSA and then incubated with DMEM/BSA containing 0.5 mM isobutylmethylxanthine (Sigma–Aldrich, St. Louis, MO, USA) at 37 °C for another 15 min. Then, different concentrations of NDP-MSH, ACTH (1–24), α-MSH, or β-MSH were added to make the final concentration of ligands ranging from 10^−12^ to 10^−6^ M for NDP-MSH and ACTH (1–24) or from 10^−11^ to 10^−5^ M for α-MSH and β-MSH. After an hour’s incubation at 37 °C, the reaction was terminated on ice and the intracellular cAMP was collected by adding 0.5 M perchloric acid containing 180 μg/mL theophylline (Sigma–Aldrich) and 0.72 M KOH/0.6 M KHCO_3_ into each well. The cAMP levels were measured by radioimmunoassay (RIA) [43].

To investigate the potential effect of caMRAP2 on caMC4R signaling, two ratios of caMC4R and caMRAP2 (1:0 and 1:5) were applied to co-transfect cells, and two ligands, α-MSH and ACTH (1–24), were used. To explore the dose-dependent effect of caMRAP2 on the maximal response (R_max_) of cAMP levels to α-MSH and ACTH (1–24) stimulation, cells were co-transfected with caMC4R and caMRAP2 in four ratios (1:0, 1:1, 1:3, and 1:5). To study the constitutive activity of G_s_ cAMP, cells were transfected with different concentrations caMC4R plasmid (0, 0.007, 0.015, 0.030, 0.060, 0.125, and 0.250 μg/μL).

### 2.11. Statistical Analysis

All data were expressed as the mean ± SEM. Statistical analysis of gene expression was performed with SPSS 19.0 software. Significant differences were calculated using one-way analysis of variance (ANOVA), followed by the post hoc test (least significant difference test and Duncan’s multiple range test), after confirming for data normality and homogeneity of variances. GraphPad Prism 6.0 software (San Diego, CA, USA) was used to calculate the parameters of ligand binding and cAMP signaling assays including B_max_, IC_50_, R_max_, and EC_50_. The significance of differences in ligand binding and signaling between caMC4R and hMC4R were determined by Student’s t-test. Parameters in flow cytometry, ligand binding, and cAMP of caMC4R regulated by MRAP2s were analyzed for significance of differences by one-way ANOVA.

## 3. Results

### 3.1. Molecular Cloning of Snakehead mc4r and mrap2 Genes

**Figure 1:**
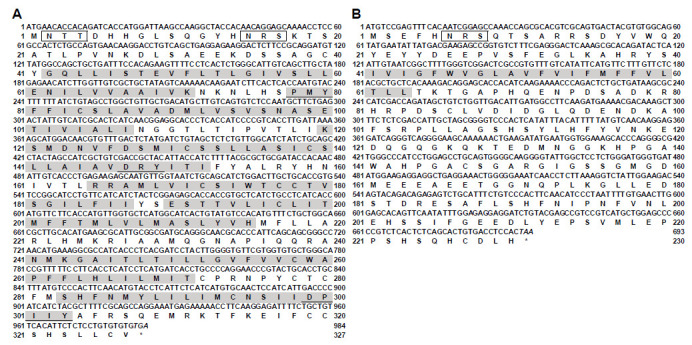
Nucleotide and deduced protein sequences of snakehead MC4R (**A**) and MRAP2 (**B**). Positions of nucleotide and amino acid sequences were indicated on both sides. Open boxes denoted the consensus sequence for N-linked glycosylation sites. Amino acids in shaded boxes indicated putative transmembrane domains. Underlines showed PMY, DRY, and DPxxY motifs in MC4R. Asterisk (*) indicated stop codon.

The cDNA sequence of snakehead *mc4r* was 1515 bp in length, and contained a 984 bp open reading frame (ORF) encoding a putative 327-amino acid protein, and has been deposited into the GenBank database (accession number KU728167) (Figure 1A).

Similar to other teleost *mc4r*, the coding region of the snakehead *mc4r* was intronless. Potential glycosylation and phosphorylation sites were found in N- and C-termini, respectively (Figure 1A and Figure S1A). In addition, cysteine residues at positions 40, 84, 131, 178, 197, 258, 272, 278, 280, and 319 were highly conserved in all vertebrate MC4Rs (Figure S1A). Alignment showed the deduced amino acid sequence of snakehead MC4R was 66% and 93% identical to human and seabass MC4Rs, respectively (Figure S1A). The sequence identity was unevenly distributed, with the N-terminal extracellular domain displaying the lowest identity to other MC4Rs, including seabass MC4R (Figure S1A).

The ORF of snakehead *mrap2* was 693 bp long, encoding a protein of 230 amino acids (Figure 1B), and has been deposited into the GenBank database (accession number MW118677). Consistent with other teleost MRAP2s, snakehead MRAP2 possessed a glycosylation site in the N-terminus, a conserved motif (LKAHKYS) crucial for the formation of antiparallel homodimer, a single TMD, and a conserved motif (NIPNFVN) in C-terminus (Figure 1B and Figure S2), with a calculated PI and molecular weight of 4.73 and 25.79 kDa, respectively. Snakehead MRAP2 showed 40.0% and 38.3% similarity with its homologs from human and chicken, and shared 42.7%, 37.5%, 55.0%, and 77.5% identity with zebrafish (MRAP2a and MRAP2b), pufferfish, and grouper MRAP2s (Figure S2).

### 3.2. Comparative Synteny of mc4r and mrap2 in Various Vertebrate Genomes

**Figure 2:**
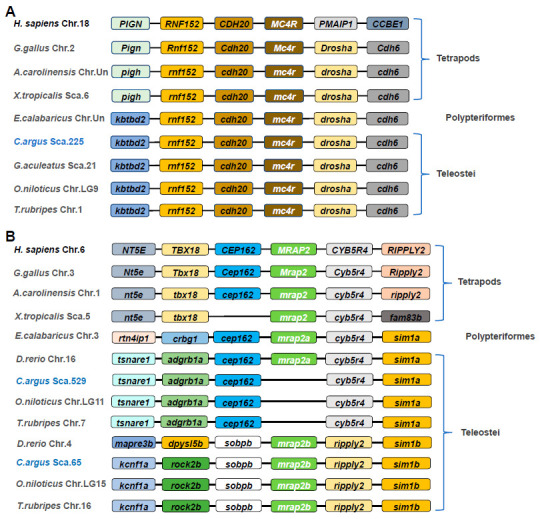
Comparative gene synteny of *MC4R* (**A**) and *MRAP2* (**B**) among representative vertebrate genomes. The colored boxes represent genes and solid lines stand for intergenic regions. The genes showing conserved synteny were indicated with same color. Taxon names of selected species were shown on the left side of the figure.

In the present study, a comparative genomic analysis was performed to investigate the genetic diversity of *MC4R* and *MRAP2* in vertebrates (Figure 2). As shown in Figure 2A, a conserved gene cluster *RNF152-CDH20-MC4R-DROSHA-CDH6* was identified in genomes of nearly all representative species, implying that the synteny of *MC4R* was highly conserved across vertebrates (Figure 2A).

Different from *MC4R*, *mrap2* possessed two isoforms in teleosts and only a single gene in tetrapods (Figure 2B). Generally, a gene conserved cluster *NT5E-TBX18-CEP162-MRAP2-CYB5R4-RIPPLY2* was identified in tetrapods, and the core gene cluster *CEP162-MRAP2a-CYB5R4* was also found in teleosts (Figure 2B), such as zebrafish (*D. rerio*) and a living fossil species reedfish (*E. calabaricus*). However, *mrap2a* was lost in Perciform fishes including snakehead (*C. argus*), tilapia (*O. niloticus*), and pufferfish (*T. rubripes*). Interestingly, a different gene cluster *SOBPb-MRAP2b-RIPPLY2-SIM1b* was identified in almost all teleosts examined in the present study, except reedfish (Figure 2B).

### 3.3. Phylogenetic Analysis

**Figure 3:**
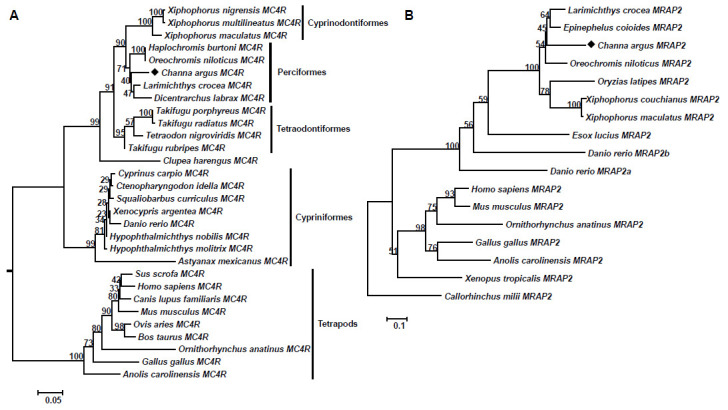
Phylogenetic trees of vertebrate MC4R (**A**) and MRAP2 (**B**). Both trees were constructed with MEGA 6.0 software based on protein datasets. The values at the nodes represented bootstrap percentages from 1000 replicates. The caMC4R and caMRAP2 were marked with a black diamond. The IDs of protein sequences used in present study were shown in Tables S2 and S3.

Two phylogenetic trees were constructed using Neighbor-Joining (NJ) method based on a series of protein sequences of vertebrate MC4Rs and MRAP2s, and both trees were robust with high bootstrap values (Figure 3).

As shown in Figure 3A, the phylogenetic tree was divided into two groups, teleost MC4Rs and higher vertebrate MC4Rs, and the teleost MC4R group could be further divided into four subgroups containing Cyprinodontiformes, Perciformes, Tetraodonformes, and Cypriniformes. Meanwhile, MC4R in Perciformes appeared posterior to Cypriniformes, but prior to Cyprinodontiformes in the evolutionary history of vertebrates (Figure 3A). Moreover, the snakehead MC4R was grouped into Perciformes, and shared a close relationship with seabass (*Dicentrarchus labrax*) and large yellow croaker (*Larimichthys crocea*) MC4Rs (Figure 3A). Additionally, the phylogenetic tree of MRAP2 was clustered into two groups of tetrapods and teleosts, and snakehead MRAP2 was grouped into the Perciforms with a close relationship with tilapia, orange-spotted grouper, and large yellow croaker (Figure 3B). Notably, it is shown that zebrafish MRAP2b shared close relationship with MRAP2 from other teleosts, in comparison with its paralog MRAP2a (Figure 3B).

### 3.4. Tissue Distribution of Snakehead mc4r and mrap2 Genes

**Figure 4:**
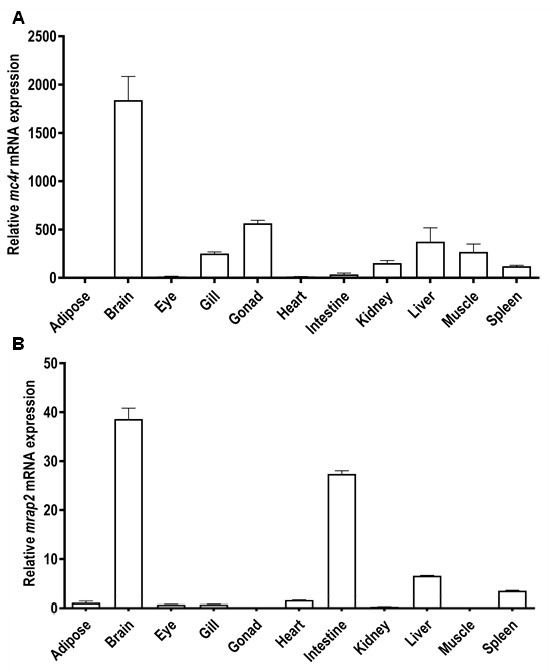
Tissue distribution patterns of snakehead *mc4r* (**A**) and *mrap2* (**B**). Eleven tissues were studied in the present study. Results were expressed as relative expression levels and standardized by *tubα1-b* gene. Error bars represented standard error of the mean (SEM) (*n* = 6).

The expression of snakehead *mc4r* and *mrap2* was determined using quantitative real-time PCR. Similar to other fish, snakehead *mc4r* was found to be extensively expressed in central and peripheral tissues, including adipose, brain, eye, gill, gonad (ovary), heart, intestine, kidney, liver, muscle, and spleen (Figure 4A), with the highest expression in the brain (Figure 4A).

Similarly, snakehead *mrap2* was also widely distributed in these tissues with the highest expression in brain (Figure 4B).

### 3.5. Effects of Fasting and Refeeding on the Expressions of Snakehead mc4r and mrap2 Genes

**Figure 5:**
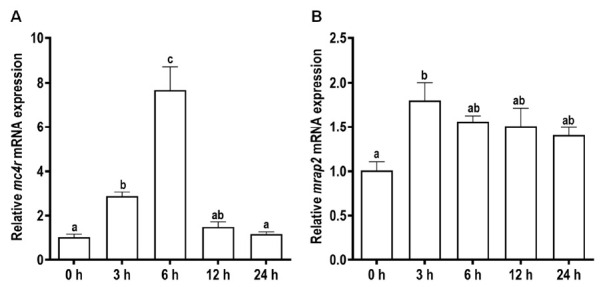
Effects of short-term (24 h) fasting on the expressions of cerebral *mc4r* (**A**) and *mrap2* (**B**) in snakehead. Data were represented as mean ± SEM (*n* = 6). Significant differences (*p* < 0.05) between groups were detected using a one-way ANOVA. Groups that differ significantly were indicated by different letters above bars.

To investigate the potential roles of snakehead MC4R and MRAP2 in energy balance, the mRNA expression levels of both genes in the brain were determined after food deprivation and refeeding. In the short-term trial, the *mc4r* mRNA level was significantly increased at 3 and 6 h while returned to basal level at 12 and 24 h after food deprivation (Figure 5A).

**Figure 6:**
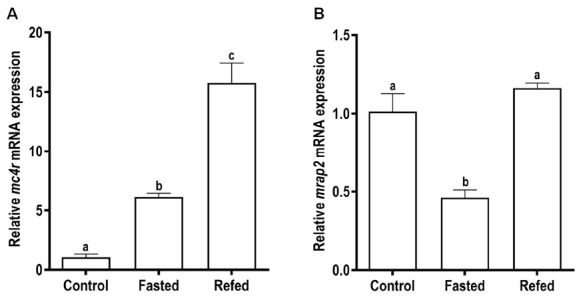
Effects of long-term (2 weeks) fasting and refeeding on expressions of brain *mc4r* (**A**) and *mrap2* (**B**) in snakehead. Data were presented as mean ± SEM (*n* = 6). Significant difference (*p* < 0.05) between groups was determined using one-way ANOVA. Groups that differ significantly were indicated by different letters above bars.

Meanwhile, the *mrap2* mRNA level was significantly increased at 3 h, and then maintained at relatively high mRNA levels at 6, 12, and 24 h (Figure 5B). In the long-term trial, the expression level of *mc4r* was significantly increased after two-week fasting, and further increased after refeeding (Figure 6A). Differently, the mRNA level of *mrap2* was dramatically decreased after fasting whereas recovered to the control level after refeeding (Figure 6B).

### 3.6. Ligand Binding Properties of caMC4R

**Figure 7:**
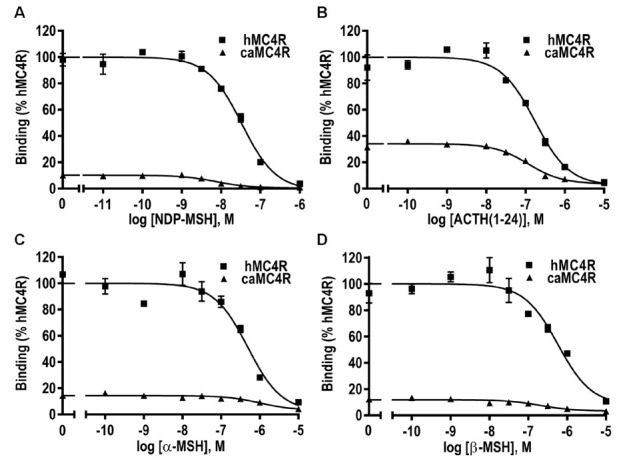
Ligand binding properties of caMC4R. HEK293T cells were transiently transfected with MC4R plasmids, and the binding properties were measured 48 h later by displacing the binding of ^125^I-NDP-MSH using different concentrations of unlabeled NDP-MSH (**A**), ACTH (1–24) (**B**), α-MSH (**C**), or β-MSH (**D**) as described in Materials and Methods. Data were expressed as % of hMC4R binding ± range in the absence of competitor from duplicate measurements within one experiment. All experiments were performed at least three times independently.

Ligand binding assays were performed to determine the binding properties of caMC4R with different MC4R ligands. Four unlabeled ligands (NDP-MSH, ACTH (1–24), α-MSH and β-MSH) were used to compete with ^125^I-NDP-MSH. The maximal binding value of caMC4R was significantly lower than that of hMC4R (16.32 ± 2.46% of hMC4R) (Figure 7 and Table 1).

When NDP-MSH or α-MSH was used to displace the ^125^I-NDP-MSH, caMC4R showed significantly decreased or increased IC_50_ values in comparison with hMC4R, whereas when the other two ligands were used as competitors, hMC4R and caMC4R exhibited similar IC_50_s (Figure 7 and Table 1).

### 3.7. Signaling Properties of caMC4R

**Figure 8:**
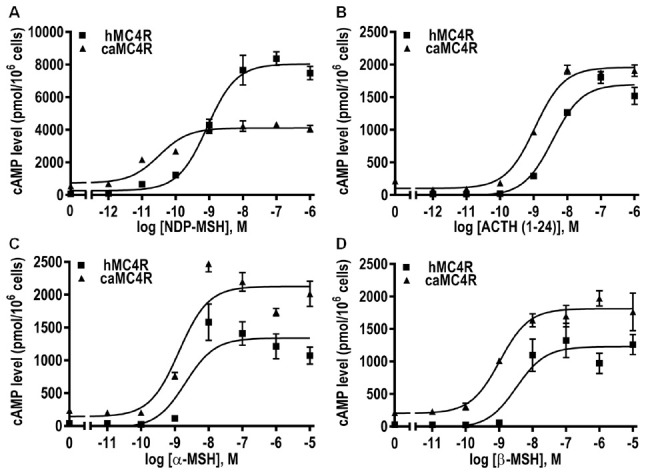
Signaling properties of caMC4R. HEK293T cells were transiently transfected with MC4R plasmids, and the intracellular cAMP levels were measured 48 h later via radioimmunoassay. Different concentrations of NDP-MSH (**A**), ACTH (1–24) (**B**), α-MSH (**C**), or β-MSH (**D**) were used to stimulate HEK293T cell. Data were expressed as mean ± SEM from triplicate measurements within one experiment. The curves were representative of at least three independent experiments.

To investigate whether caMC4R could respond to ligand stimulation with increased cAMP level, RIA was carried out. As shown in Figure 8, all ligands could increase the intracellular cAMP level in a dose-dependent manner. The EC_50_ of ACTH (1–24) for caMC4R was significantly lower than that of hMC4R, whereas the EC_50_s of NDP-MSH, α-MSH, and β-MSH were comparable with those of hMC4R (Table 2). The caMC4R showed higher maximal responses than hMC4R when stimulated with ACTH (1–24), α-MSH, and β-MSH, and slightly decreased maximal response in response to NDP-MSH (Figure 8 and Table 2).

**Figure 9:**
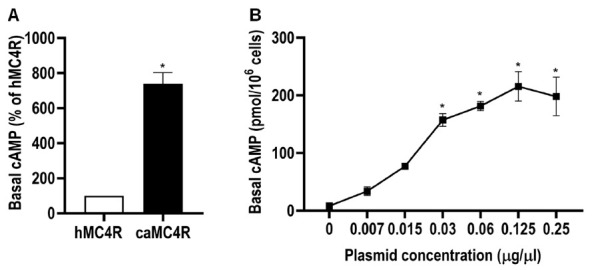
Constitutive activities of caMC4R in cAMP signaling pathway. HEK293T cells were transiently transfected with 0.25 µg/µL hMC4R or caMC4R to compare their basal cAMP activity (**A**) or transfected with increasing concentrations of caMC4R (**B**). Data were expressed as mean ± SEM (*n* ≥ 3). Asterisk (*) showed significant difference (*p* < 0.05) of basal cAMP level compared with respective control group (Student’s *t*-test in A and one-way ANOVA followed by Tukey-test in B).

The basal cAMP level of caMC4R was also measured. The results demonstrated caMC4R had significantly increased basal activity, which was 7.39 times that of hMC4R (Figure 9A and Table 2). To confirm constitutive activity of caMC4R, different concentrations of caMC4R plasmids were transfected into HEK293T cells and basal cAMP levels were measured. The high basal intracellular cAMP level was detected at only 0.03 μg/μL caMC4R plasmid transfected (Figure 9B).

### 3.8. Modulation of caMC4R Expression and Pharmacological Properties by caMRAP2

**Figure 10:**
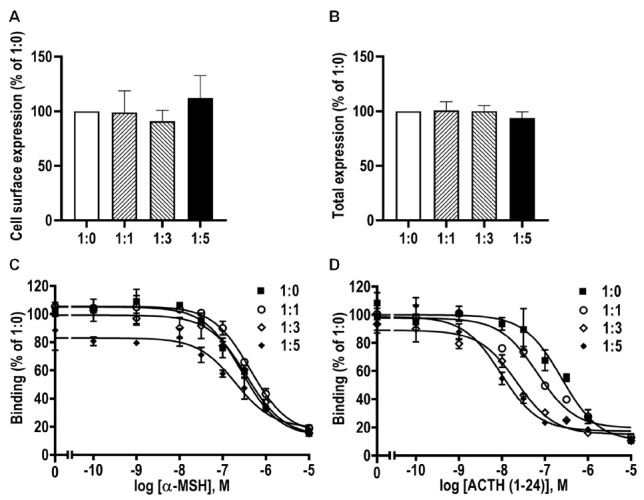
Modulation of caMC4R expression and binding properties by caMRAP2. HEK293T cells were co-transfected with caMC4R/caMRAP2 in four different ratios (1:0, 1:1, 1:3, and 1:5). Cell surface (**A**) and total (**B**) expression levels of caMC4R were measured by flow cytometry. Ligand binding experiments (**C**,**D**) were performed as in Figure 7. The results, calculated as percentage of 1:0 group, were expressed as the mean ± SEM of at least three independent experiments.

To investigate the effects of caMRAP2 on the cell surface and total expression levels of caMC4R, flow cytometry was performed. No significant difference was observed among the four groups for cell surface or total expression (Figure 10A,B). Competitive ligand binding assays showed that IC_50_s of caMC4R to ACTH (1–24) was decreased by caMRAP2 in the 1:3 and 1:5 groups, whereas no change was observed in IC_50_s for α-MSH among the four groups (Figure 10C,D and Table 3).

**Figure 11:**
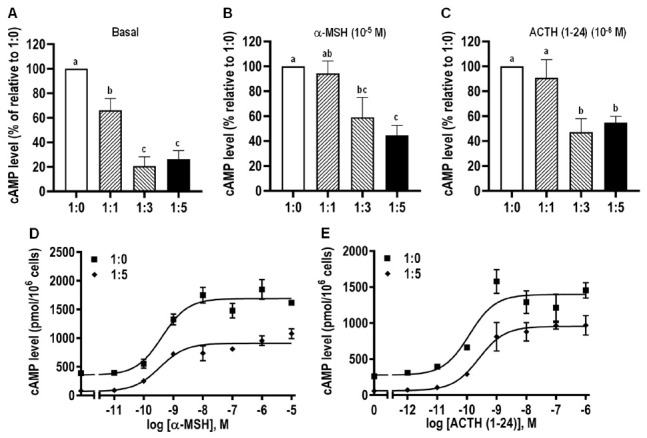
Effects of caMRAP2 on signaling properties of caMC4R. HEK293T cells were co-transfected with caMC4R/caMRAP2 in four different ratios (1:0, 1:1, 1:3, and 1:5), and cAMP levels under basal (**A**) or stimulated with 10^−5^ M α-MSH or 10^−6^ M ACTH (1–24) (**B**,**C**) were measured. The results were calculated as a percentage of 1:0 group. Results were expressed as mean ± SEM (*n* ≥ 3). Different letters indicate significant difference (one-way ANOVA followed by Tukey-test). (**D**,**E**) The curves represented at least three independent experiments in which different concentrations of α-MSH or ACTH (1–24) were used to stimulate cells co-transfected with caMC4R and caMRAP2 at the ratios of 1:0 and 1:5.

In order to explore the effect of caMRAP2 on the cAMP signaling of caMC4R, cells were co-transfected with caMC4R and caMRAP2 in different ratios. The results showed that caMRAP2 dose-dependently decreased the basal cAMP levels (Figure 11A). In addition, caMRAP2 significantly reduced signaling induced by both α-MSH and ACTH (1–24) (Figure 11B,C and Table 4). Dose–response experiments showed that caMRAP2 only decreased the maximal response with no effect on EC_50_s (Figure 11D,E and Table 4).

## 4. Discussion

In this study, we cloned snakehead *mc4r* and *mrap2* genes. Sequence comparison showed that caMC4R shared more than 80% amino acid identity with other teleost MC4Rs, whereas caMRAP2 shared lower identity (37.5–77.5%) with other teleost MRAP2s, suggesting MC4R was more conserved than MRAP2 in teleosts.

Comparative gene synteny analysis showed that only a single copy of *mc4r* was identified in vertebrates with a conservative gene cluster *RNF152-CDH20-MC4R-DROSHA-CDH6*, suggesting that this receptor is highly conserved and may play similar roles in vertebrates. In tetrapod, only a single copy of *mrap2* was identified in their genomes, exhibiting a conserved gene cluster *NT5E-TBX18-CEP162-MRAP2-CYB5R4-RIPPLY2*. In teleosts, the gene number of *mrap2* is variable. In zebrafish, two copies of *mrap2* (*mrap2a* and *mrap2b*) were identified, and functional studies showed that MRAP2a decreases binding affinity for α-MSH while MRAP2b increases ligand sensitivity of MC4R [35]. In reedfish, only a single *mrap2* was identified and it shares a similar conserved genetic locus with tetrapod *mrap2* and zebrafish *mrap2a*, suggesting that the duplication of *mrap2* might have occurred after the specific whole genome duplication event in teleost [55]. Interestingly, we found that the homolog of zebrafish *mrap2a* was lost in Perciform and Tetraodontiform fishes, including snakehead, tilapia, and puffer fish. However, the *mrap2b* paralog was retained in these teleost genomes with a conserved gene cluster *SOBPb-MRAP2b-RIPPLY2-SIM1b*, suggesting the evolutionary process of *mrap2* is complex in teleosts and the physiological role of this gene might be variable.

To investigate the evolutionary relationship of vertebrate MC4Rs and MRAP2s, phylogenetic trees were constructed. Both trees were divided into two subgroups of teleost and tetrapod subgroup, similar to previous studies [28,32,33]. For the MC4R tree, the Cypriniformes clade was located at the root of the subgroup, suggesting the Cypriniformes MC4R might have appeared earlier in the evolutionary history than other teleosts. Meanwhile, the snakehead MC4R was clustered into Perciformes clade, and shared a close relationship with seabass MC4R. These findings were consistent with the traditional species classification [27,28]. For the MRAP2 tree, zebrafish MRAP2b shares a close relationship with MRAP2 from other teleosts, in comparison with its paralog MRAP2a, suggesting the restrained MRAP2 in snakehead and other teleosts is the homolog of zebrafish MRAP2b, consistent with gene synteny analysis.

Data from real-time quantitative PCR showed that *mc4r* was widely expressed in various tissues with high expression in brain and gonad, similar to previous studies [27,28,29,30,31,32,33,56], suggesting that snakehead MC4R might also be involved in regulating food intake [25,32] and sexual maturation [12,57]. Meanwhile, *mrap2* was also extensively distributed in several tissues, with relatively high expression in brain and gonad. This distribution pattern of *mrap2* was also found in orange-spotted grouper [30], Nile tilapia [39], and topmouth culter [31], suggesting functional identity of MRAP2 in teleosts. Additionally, *mc4r* and *mrap2* exhibited similar distribution pattern, indicating that they might interact with each other in snakehead.

We measured *mc4r* and *mrap2* expression after short-term and long-term fasting and refeeding. We showed that expression of these genes was modulated by these manipulations (Figure 5 and Figure 6), indicating their potential involvement in regulating energy homeostasis. Several previous studies also investigated the expression of these genes with fasting and refeeding, obtaining conflicting results in different species [32,33,56,58,59]. Further studies, including physiological experiments involving measuring energy balance with in vivo administration of ligands, are needed to clarify the physiological function of MC4R.

To investigate the pharmacological characteristics of caMC4R, four ligands were used, together with hMC4R for comparison. Our results demonstrated that caMC4R was fully functional. The synthetic agonist NDP-MSH bound to caMC4R with the highest affinity with an IC_50_ of 4.50 nM, which is about 7-fold lower than hMC4R. Additionally, it stimulated caMC4R with the highest potency with an EC_50_ of 0.04 nM. α- and β-MSHs stimulated caMC4R with similar potency as hMC4R. However, ACTH (1–24) had 3-fold higher potency at caMC4R than hMC4R. Previous studies demonstrated that fish MC4Rs have decreased binding capacities [27,28,29,30,31,60]. We also found lower binding capacity in caMC4R (16.32% of that of hMC4R) (Figure 7 and Table 1). Among the three endogenous ligands used here, ACTH (1–24) showed high binding affinity, further supporting the suggestion that ACTH (1–24) might act as the ancestral ligand for MCRs.

A remarkably elevated basal cAMP activity was found in caMC4R. This phenomenon has also been reported in other fish species, such as spotted scat [27], grass carp [28], swamp eel [29], orange-spotted grouper [30], and topmouth culter [31]. In contrast to hMC3R, hMC4R has some basal activity [61]. Srinivasan and colleagues reported that part of the N-terminus functions as a tethered intramolecular ligand to maintain the constitutive activity of hMC4R [62]. Here, low sequence homology was found within the N-terminus in caMC4R when compared with hMC4R. We hypothesized that structural difference of N-termini may account for its high basal activity. The loss of constitutive activity in mutant hMC4Rs is one cause of obesity [62,63]. Therefore, the development of an MC4R agonist has been regarded as a therapeutic option for human obesity treatment. However, increasing food intake and decreasing energy expenditure of aquaculture species will deliver benefits for farmers. As a result, focusing on the study of caMC4R antagonists will promote the development of snakehead cultivation.

In this study, we showed that caMRAP2 did not affect cell surface and total expression of caMC4R, similar to the observation in chicken [59], but different from results obtained in tilapia [39] and mouse [22] (decreased cell surface expression), or zebrafish [35] and topmouth culter [31] (increased cell surface expression). Therefore, MRAP2 has a differential effect on MC4R membrane expression in different species. Snakehead MRAP2 increased binding affinity of caMC4R to ACTH (1–24), but did not affect the IC_50_ of caMC4R to α-MSH. In topmouth culter, two isoforms of MRAP2 are also shown to increase binding affinity of MC4R to ACTH (1–24), but not to α-MSH [31]. In addition to its chaperoning role, MRAP1 also enables MC2R to form the high-affinity binding pocket to ACTH [64]. MRAP2 might also make caMC4R to achieve a conformation with enhanced affinity to ACTH. The high constitutive activity in cAMP pathway of caMC4R was inhibited by caMRAP2, similar to our previous observations in orange-spotted grouper [30] and topmouth culter [31]. In the presence of caMRAP2, significantly decreased maximal response but not EC_50_ of caMC4R in response to α-MSH or ACTH (1–24) was observed. Previous studies demonstrated that MC4R can become an ACTH receptor by increasing ligand sensitivity to ACTH in the presence of MRAP2 [36,59,65]. In our experiments, ligand sensitivity to ACTH (1–24) of caMC4R was not increased by caMRAP2, despite the increased binding affinity to ACTH (1–24) by caMRAP2.

In addition to energy homeostasis, MC4R also regulates reproduction and sexual function [2]. Compared with mammals, the evidence of MC4R in regulating teleost reproduction is relatively scarce. In swordtail fish, nonfunctional Y-linked *mc4r* copies in larger males act as dominant-negative mutants and delay puberty onset [57]. In spotted scat, MC4R stimulates *gnrh* expression in the hypothalamus, thereby modulating pituitary FSH and LH synthesis [66]. In black rockfish, α- and β-MSHs increase brain *gnih* and decrease *sgnrh* and *cgnrh* expression in vitro; they also affect expression of some ovarian genes. These studies demonstrated that MC4R could play a vital role in reproductive regulation in fish. In the present study, we also observed that *mc4r* and *mrap2* were highly expressed in gonad, implying a potential role in reproduction. Therefore, future studies on MC4R regulation of reproductive function are warranted.

## 5. Conclusions

In conclusion, our studies on snakehead MC4R and MRAP2 and their interaction contributes to a better understanding of fish MC4Rs. Some aspects, such as high expression of these genes in the brain and significant expression in the gonad, and high basal activity were similar to previous studies in fish. However, our data, combined with literature, suggested that functional implications of MRAP2 on MC4R are divergent in different species. Whether MC4R could be modulated to improve growth and reproduction of snakehead remains to be investigated.

## Figures and Tables

**Table 1:** The binding properties of caMC4R.

MC4R	B_max_ (%)	NDP-MSH	ACTH (1–24)	α-MSH	β-MSH
		IC_50_ (nM)	IC_50_ (nM)	IC_50_ (nM)	IC_50_ (nM)
hMC4R	100	27.72 ± 4.29	324.6 ± 88.97	488.2 ± 2.55	749.4 ± 93.98
caMC4R	16.32 ± 2.46 ^c^	4.50 ± 1.07 ^a^	415.1 ± 163.7	981.0 ± 45.52 ^b^	328.7 ± 119.6

Results were expressed as the mean ± SEM of at least three independent experiments. ^a^ Significantly different from the parameter of hMC4R, *p* < 0.05. ^b^ Significantly different from the parameter of hMC4R, *p* < 0.01. ^c^ Significantly different from the parameter of hMC4R, *p* < 0.0001.

**Table 2:** The signaling properties of caMC4R.

MC4R		hMC4R	caMC4R
Basal (%)		100	739.2 ± 63.82 ^b^
NDP-MSH	EC_50_ (nM)	0.59 ± 0.18	0.04 ± 0.02
	R_max_ (%)	100	75.18 ± 27.83
ACTH (1–24)	EC_50_ (nM)	2.68 ± 0.50	0.81 ± 0.18 ^a^
	R_max_ (%)	100	119.0 ± 3.73 ^a^
α-MSH	EC_50_ (nM)	2.82 ± 0.40	2.32 ± 0.57
	R_max_ (%)	100	198.1 ± 55.95
β-MSH	EC_50_ (nM)	6.67 ± 3.41	2.49 ± 0.81
	R_max_ (%)	100	163.9 ± 89.36

Results were expressed as the mean ± SEM of at least three independent experiments. ^a^ Significantly different from the parameter of hMC4R, *p* < 0.05. ^b^ Significantly different from the parameter of hMC4R, *p* < 0.01.

**Table 3:** The effect of caMRAP2 on ligand binding properties of caMC4R.

caMC4R/caMRAP2	B_max_ (%)	α-MSH	ACTH (1–24)
		IC_50_ (nM)	IC_50_ (nM)
1:0	100	383.80 ± 68.82	239.90 ± 23.61
1:1	100.70 ± 6.24	461.00 ± 65.51	144.80 ± 74.73
1:3	102.80 ± 9.56	497.10 ± 147.4	49.48 ± 19.47 ^a^
1:5	83.62 ± 7.17	238.10 ± 60.77	24.73 ± 11.73 ^a^

Results were expressed as the mean ± SEM of at least three independent experiments. ^a^ Significantly different from the parameter of 1:0, *p* < 0.05.

**Table 4:** The effect of caMRAP2 on signaling properties of caMC4R.

caMC4R/caMRAP2	α-MSH		ACTH (1–24)	
	EC_50_ (nM)	R_max_ (%)	EC_50_ (nM)	R_max_ (%)
1:0	0.48 ± 0.12	100	0.45 ± 0.20	100
1:5	0.61 ± 0.15	44.70 ± 7.96 ^c^	0.42 ± 0.13	54.81 ± 5.19 ^c^

Results are expressed as the mean ± SEM of at least three independent experiments. ^c^ Significantly different from the parameter of 1:0, *p* < 0.001.

## Data Availability

The raw data supporting the conclusions of this article will be made available by the authors upon request, without undue reservation.

## Supplementary Materials

The following are available online at https://www.mdpi.com/2218-273X/11/3/481/s1.
