# Supplementary material for: MRAP2 Interaction with Melanocortin-4 Receptor in SnakeHead (Channa argus)
Source: Biomolecules. 2021 Mar 23;11(3):481. doi: 10.3390/biom11030481 (PMC8004712; doi:10.3390/biom11030481)
Supplement: Supplementary file 1 [file biomolecules-11-00481-s001.pdf]

biomolecules

Article

MRAP2 Interaction with Melanocortin-4 Receptor in Snakehead (*Channa argus*)

Zheng-Yong Wen [1,2,3,*,†], Ting Liu [4,†], Chuan-Jie Qin [1,2], Yuan-Chao Zou [1,2], Jun Wang [1,2], Rui Li [1,2] and Ya-Xiong Tao [4,*]

[1] Key Laboratory of Sichuan Province for Fish Conservation and Utilization in the Upper Reaches of the Yangtze River, Neijiang Normal University, Neijiang, Sichuan 641100, China; qinchuanjie@126.com (C.-J.Q.); zou3891@163.com (Y.-C.Z.); wangjunzl@126.com (J.W.); liruitiandi@sina.com (R.L.)
[2] College of Life Science, Neijiang Normal University, Neijiang, Sichuan 641100, China; qinchuanjie@126.com (C.J.Q.); zou3891@163.com (Y.C.Z.); wangjunzl@126.com (J.W.); liruitiandi@sina.com (R.L.)
[3] BGI Education Center, University of Chinese Academy of Sciences, Shenzhen 518083, China
[4] Department of Anatomy, Physiology and Pharmacology, College of Veterinary Medicine, Auburn University, Auburn, AL 36849, United States; tzl0057@auburn.edu (T.L.)
† These authors contributed equally.
* Correspondence: zhengyong_wen@126.com; Tel.: +86 18582681220 (Z.Y.W.); taoyaxi@auburn.edu; Tel.: +1 3348445396 (Y.X.T.)

Citation: Wen, Z.-Y.; Liu, T.; Qin, C.-J.; Zou, Y.-C.; Wang, J.; Li, R.; Tao, Y.-X. MRAP2 Interaction with Melanocortin-4 Receptor in Snakehead (*Channa argus*). **2021**, *11*, 481. https://doi.org/10.3390/biom11030481

Academic Editor: Paolo Annibale

Received: 26 January 2021
Accepted: 19 March 2021
Published: 23 March 2021

Publisher's Note: MDPI stays neutral with regard to jurisdictional claims in published maps and institutional affiliations.

Copyright: © 2021 by the authors. Licensee MDPI, Basel, Switzerland. This article is an open access article distributed under the terms and conditions of the Creative Commons Attribution (CC BY) license (http://creativecommons.org/licenses/by/4.0/).

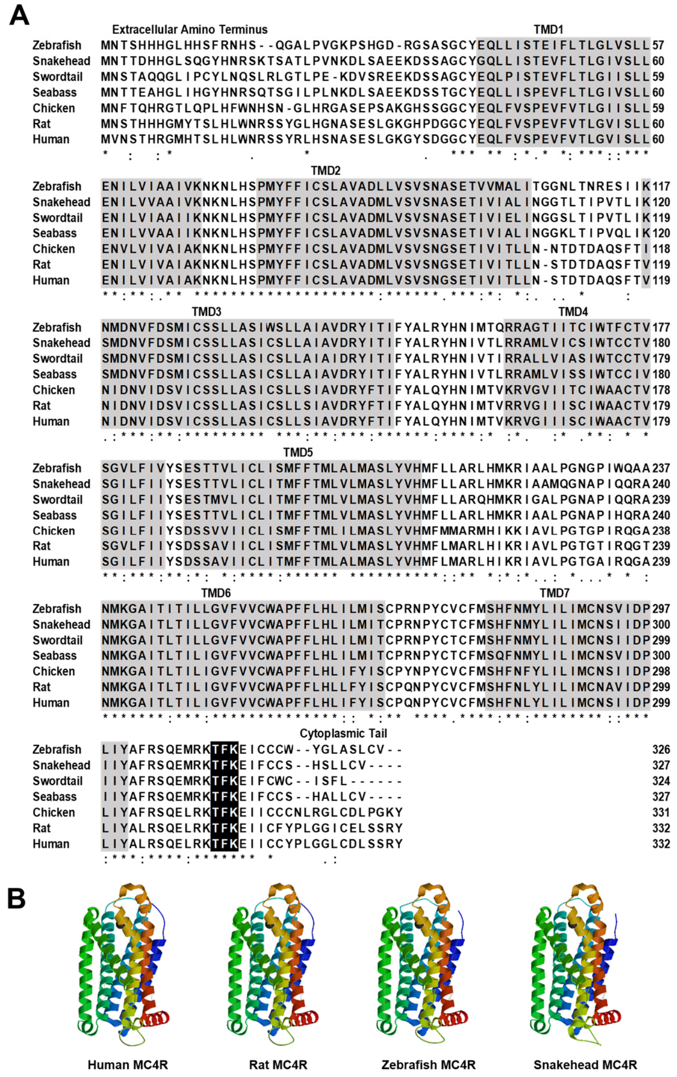

Figure S1. Comparison of amino acid sequences between caMC4R and MC4Rs from other species (A) and the putative three-dimensional structure of snakehead MC4R and those of relative model species (B). Labeled as follows: trans-membrane domains were shown in shaded boxes and named as TMD 1-7, amino and carboxyl termini were represented as extracellular amino terminus and cytoplasmic tail, respectively. Predicted phosphorylation sites were shown by dark shadow. Asterisk (*) indicated the same amino acids. Cellular was shown by a green frame box, followed with an extracellular N-terminus and intracellular C-terminus, respectively. Seven transmembrane domains were shown in the figure. Amino acid was represented by a circular round.

```
                                                                      TMD
Human         MSAQRLISNRTSQQSASNSDYTWEYEYYEIG-PVSFEGLKAHKYSIVIGFWVGLAVFVIF 59
Chicken       MSALRLISNRTSQQALSNSDYTWEYEYYEYG-PVSFEGLKAHKYSIVIGFWVGLAVFVIF 59
Snakehead     MSEF----HNRSQTSARRSDYVWQYEYYD-EEPVSFEGLKAHRYSIVIGFWVGLAVFVIF 55
Grouper       MSDF----HNRSQTSARRSDYVWQYEYYDDEEPVSFEGLKAHRYSIVIGFWVGLAVFVIF 56
Fugu          MSAR----GNRSQSSARRGDYIWQYEYYDYDEPVSFEGLRAHRYSIVIGFWVGLAVFVIF 56
Zebrafish_2a  MPRF----QLSNSTSVPNHNYEWSYEYYDDEEPVSFEGLKAHRYSIVIGFWVGLAVFVIF 56
Zebrafish_2b  MS------EYS-NRSQAGADYEWHYEYYEDEEPVSFEGLRANRYSIVIGFWVGLAVFVIF 53
              *.         .  :   : * * ****:   *******:*::*****************

Human         MFFVLTLLTKTGAPHQDNAESSEKRFRMNSFVSDFGRPLE--PDKVFSR-QGNEESRSLF 116
Chicken       MFFVLTLLTKTGAPHQENTESSEKRFRMNSFVADFGRPLE--SERVFSR-QIAEESRSLF 116
Snakehead     MFFVLTLLTKTGAPHQENPDSADKRHRPDSCLVDIDGLQD-ENDKAFSR-PLLAGSHSYL 113
Grouper       MFFVLTLLTKTGAPHQENPDSAEKRHRPGSCLVDIGSPQD-ENDKAFSR-PLLAESRSYF 114
Fugu          MFFVLALLSKTRAPRQENPESADKHHRPDGYPVDIDSLQD-EKAPSFTH-PLLSESRAYS 114
Zebrafish_2a  MFFVLTLLTKTGAPHPEAAEPYEKRMRLTSCADGLGRQRETDGRTGLSR-PLLEESRSLF 115
Zebrafish_2b  MFFVLTLLTKTGAPHPEMCDASMKPHVLIGCELEVG------GSLAFSLPPLPDQSRSLF 107
              *****:**:** **: :  :.  *     .    ..          ::        *::

Human         HCYINEVERLDRAKACHQTTALDSDVQLQEAI-----RS--------------------- 150
Chicken       HFCINEVEHLDKAQQSQKGPDLESNIHFQEVS-----RS--------------------- 150
Snakehead     HFYVNKEDQGQGKQKTEDMNGGKHPGAWAHPGACSGARGIGSSGMG-DMEEEAEETGGNQ 172
Grouper       HFYINEEDQGQGKQKPGDERVGKHCGARGQQ----GTRGISSSGMMDEMEEDAEEARGHQ 170
Fugu          HFCIQN-SQDSGKKTSEDSRFGEQNLAPQVS---CHHLGGGSSPRRDTVEVDLEGVVSKQ 170
Zebrafish_2a  HCYINEEEREGGRAATDAGALTHGRSGIGNS------RG------------QVEEVG--L 155
Zebrafish_2b  HFYIHKEER--VKTHKDA---VIGR-GMHCG------RG------------NAE------ 137
              *  ::: .:                             .

Human         -------SGQPEEELNRLMKFDIPNFVNTDQN-YFGEDDLLISEPPIVLETKPLSQTSHK 202
Chicken       -------SGTLEEDLNCLAKYNIPNFVNTEQNSSLGEGDLLILQPPRVLESKTAMQSSHR 203
Snakehead     PLKGLLEDS-TDRESAFLSHFNIPNFVNLEHSSIFGEED-LYEPSV-MLEPPSHSQ--HC 227
Grouper       PLKGLIEEGKTDRECAFFSHFNIPNFVNLEHSSTLGEDDLLYEPSA-ILERQSHSQDAHC 229
Fugu          PLHGRAGDVTAEDDYVFFTQFNIPNFVSLEQSSELGEEELLYEPSA-VLER----REAHC 225
Zebrafish_2a  VVQNMVLESRAEREAALLAHFNIPNFVNSELNSALGDEDLLLGDPP-IIME--------- 205
Zebrafish_2b  ---------RADEDEHFMSSFNIPNFVNSEQSSSLGHDDFLLSEPP-IITDGQSDELKTA 187
                         : :   :  ::*****. : .  :*. : *   .  ::

Human         DLD---------                                                 205
Chicken       ILD---------                                                 206
Snakehead     DLH---------                                                 230
Grouper       DIR---------                                                 232
Fugu          DIH---------                                                 228
Zebrafish_2a  ------------                                                 205
Zebrafish_2b  EPAHLCYDIIRH                                                 199
```

Figure S2. Multiple alignment of snakehead MRAP2 with that of other species. The transmembrane domain (TMD) was boxed. The above solid line showed the conserved motif (LKAHRYS) required for the formation of antiparallel homodimer. The above dashed line denoted the conserved motif (NIPNFVN) in C-terminus. Asterisk (*) indicated the same amino acids.

Table S1. PCR primers used for cloning and gene expression studies.

Primers	Primer sequence (5′- 3′)
mc4r-F1	GTTCCTGCTCGCTGTTAA
mc4r-R1	CACGTTGTCCATGCTTTT
mc4r-F2	CGTGTTTGACTCTATGAT
mc4r-R2	TTTTACTTGGAGATTGTA
mrap2-F1	TGTTTTCAGATAGGCTTCG
mrap2-R1	ATCACCCATCCCAGAGG
mrap2-F2	TTCTTTGTTCTCACGCTG
mrap2-R2	TCTGTGCTTTATCTGTTCC
mc4r-qF	TGCCAGTGAACAAGGACC
mc4r-qR	AGCAGCGACAACCAAGAT
mrap2-qF	TGTTCTCACGCTGCTCA
mrap2-qR	GCTTTGTCGTTTTCATCT
Tubα1-qF	AGCCTGATGGTCAAATGC
Tubα1-qR	TTCCAATGGTGTAGTGCC

Table S2. Listing of MC4R sequences used in this study. Protein IDs are given to allow access to the protein sequence on Ensembl or GenBank website.

Number	*Species*	Protein ID
1	*Xiphophorus nigrensis*	ADO60278
2	*Xiphophorus maculatus*	NP_001303841
3	*Xiphophorus multilineatus*	ADO60279
4	*Haplochromis burtoni*	NP_001274332
5	*Oreochromis niloticus*	ENSONIP00000025763
6	*Channa argus*	AMM02541
7	*Larimichthys crocea*	XP_019120241
8	*Dicentrarchus labrax*	CBN82190
9	*Takifugu porphyreus*	BAB71733
10	*Takifugu rubripes*	AAO65551
11	*Takifugu radiatus*	BAB71732
12	*Tetraodon nigroviridis*	AAQ55178
13	*Clupea harengus*	XP_012679593
14	*Cyprinus carpio*	CBX89936
15	*Ctenopharyngodon idella*	AOZ60534
16	*Squaliobarbus curriculus*	ADV40875
17	*Xenocypris argentea*	ADV40878
18	*Danio rerio*	NP_775385
19	*Hypophthalmichthys molitrix*	ADV40873
20	*Hypophthalmichthys nobilis*	ADV40874
21	*Astyanax mexicanus*	ENSAMXP00000027055
22	*Sus scrofa*	ABD28176
23	*Homo sapiens*	NP_005903
24	*Canis lupus familiaris*	EDL09662
25	*Mus musculus*	EDL09662
26	*Ovis aries*	ACC77651
27	*Bos taurus*	ACR43465
28	*Ornithorhynchus anatinus*	XP_001505445
29	*Gallus gallus*	AAT73771
30	*Anolis carolinensis*	XP_003226797

Table S3. Listing of MRAP2 sequences used in this study. Protein IDs are given to allow access to the protein sequence on Ensembl or GenBank website.

Number	Species	Protein ID
1	*Homo sapiens*	NP_001333471.1
2	*Mus musculus*	NP_001346884.1
3	*Ornithorhynchus anatinus*	XP_028903035.1
4	*Gallus gallus*	NP_001307836.1
5	*Anolis carolinensis*	XP_008119910.1
6	*Xenopus tropicalis*	XP_002933963.1
7	*Channa argus*	Present study
8	*Callorhinchus milii*	XP_007906624.1
9	*Danio rerio*	XP_001342923.4
10	*Danio rerio*	XP_005168578.1
11	*Epinephelus coioides*	MK425026.1
12	*Esox lucius*	XP_010888023.1
13	*Larimichthys crocea*	XP_027140224.1
14	*Oreochromis niloticus*	XP_003458293.2
15	*Oryzias latipes*	XP_004083625.1
16	*Xiphophorus couchianus*	XP_027897067.1
17	*Xiphophorus maculatus*	XP_005813802.1
